# Absorption coefficient of water vapor across atmospheric troposphere layer

**DOI:** 10.1016/j.heliyon.2019.e01145

**Published:** 2019-01-23

**Authors:** Peng-Sheng Wei, Hsuan-Han Chiu, Yin-Chih Hsieh, Da-Lun Yen, Chieh Lee, Yi-Cheng Tsai, Te-Chuan Ting

**Affiliations:** Department of Mechanical and Electro-Mechanical Engineering, National Sun Yat-Sen University, Kaohsiung 80424, Taiwan, ROC

**Keywords:** Atmospheric science, Geophysics, Geoscience

## Abstract

Absorption coefficient of water vapor proposed to be responsible for an increase in temperature in the troposphere layer with altitude less than 10 km is systematically presented in this work. Since global warming plays an important role in affecting the human life, a confirmative and detailed study of global warming is essentially need. Solar irradiation within short wavelength range can be extinguished from absorption and scattering by the atmosphere, and absorbed and reflected by the Earth's surface. Radiative within high wavelength range from the Earth's surface can be absorbed by atmospheric water vapor, carbon dioxide and other gases. The difference in solar irradiation and energy escaped to the space from the atmosphere results in the atmosphere acting as the glass of a greenhouse and increase atmospheric temperature. Extending the previous work [1] for predicting absorption coefficient of carbon dioxide through the troposphere, this work further determines absorption coefficients of water vapor in different wavelength bands centered at 71, 6.3, 2.7, 1.87 and 1.38 μm across the temperature, pressure and concentration-dependent troposphere layer. Solving one-dimensional unsteady heat conduction-radiation equation with the COMSOL computer code, the predicted temperature together with water vapor density for different optical path lengths can be used to interpret in details absorption coefficient or the ratio between band intensity and effective band width by using the exponential wide band model. The results show that absorption coefficients are strongly affected by water vapor concentration. For example, absorption coefficients in the band centered at 71 μm increases from 0.3 to 1.2 ${\text{m}}^{-1}$ at the tropopause and 0.6 to 3.1 ${\text{m}}^{-1}$ at the Earth's surface as mole fraction of water vapor increases from 0.005 to 0.02. The predicted absorption coefficients agree with experimental and theoretical results in the literature. A more detailed and realistic temperature profile through the troposphere with optical path length of $10^{4}$ m is presented.

## 1 Introduction

**Fig. 1:**
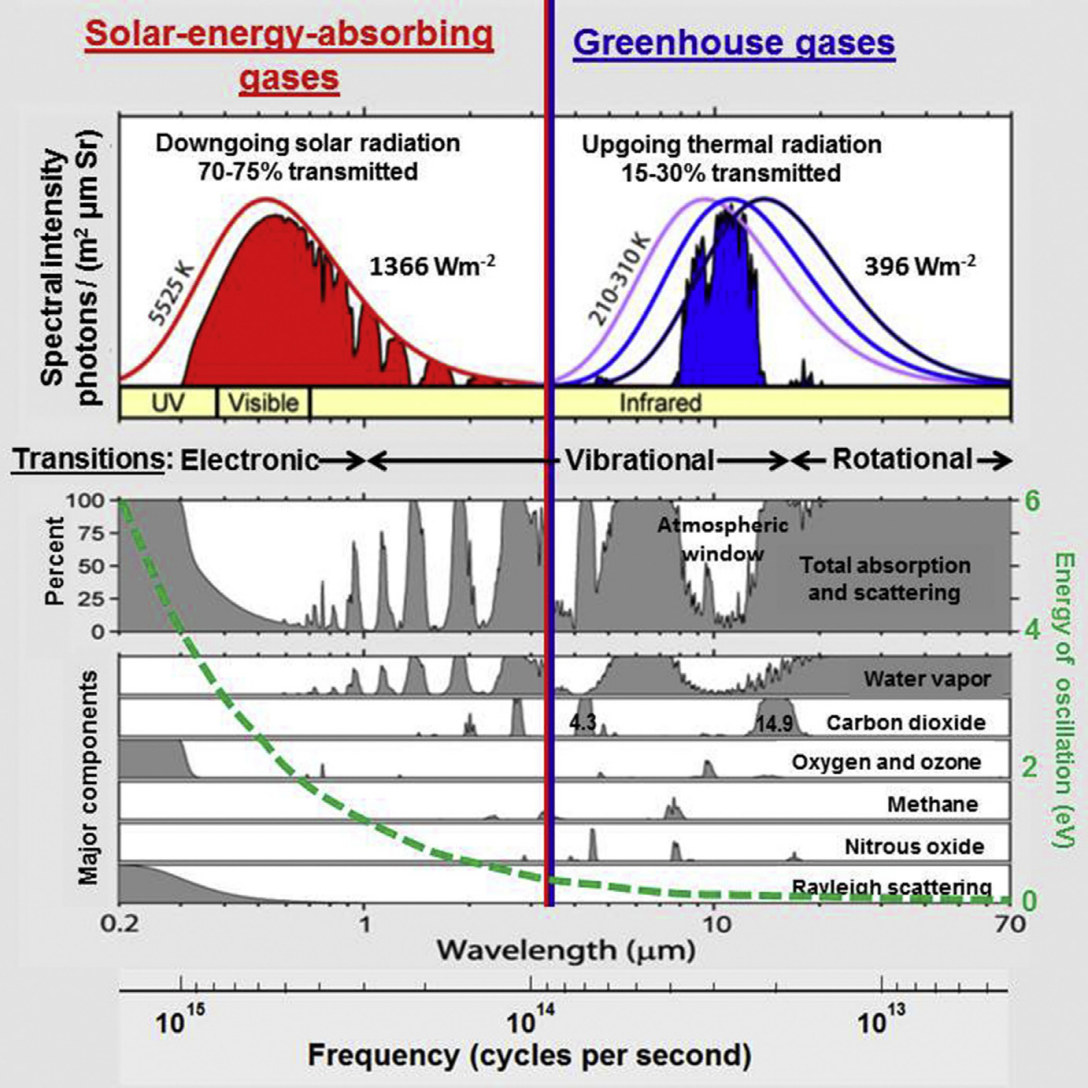
Transmission of shortwave solar irradiation and long wavelength radiation from the Earth's surface through atmosphere, as permitted by Rohde [2].

Global warming is considered to be a consequence of the greenhouse effects of water vapor and other gases such as carbon dioxide and methane, and so on. A fundamental study of radiative properties of greenhouse gases is therefore critical [1]. It can be clearly seen from Fig. 1 [2] by examining Rayleigh scattering and absorption of solar irradiation and absorption spectrum of the major greenhouse gases for upgoing thermal radiation in the Earth's atmosphere. Radiation from the Sun closely follows a blackbody spectrum at a temperature of around 5500 K. Emissions from the Earth at low temperature around a temperature of 300 K are in the infrared region. Water vapor is the most significant greenhouse gas, followed by carbon dioxide and other minor greenhouse gases. Infrared absorption and emission of radiation of polyatomic molecules such as water vapor and carbon dioxide are resulting from coupled vibrational and rotational energy transitions. Symmetric diatomic molecules such as oxygen gas and nitrogen gas have no permanent dipole moment, leading to transparent to infrared radiation. In view of extinction of Rayleigh scattering and absorption, a rough amount of 70 % of direct sunlight at the top of atmosphere passes through the atmosphere to Earth's surface. The greenhouse gases, however, absorb around 70 % of upgoing thermal radiation from the Earth's surface. In view of the difference between the downward solar irradiation and upgoing thermal radiation at the top of the atmosphere, the global warming occurs in the presence of greenhouse gases.

Water vapor contributes significantly to the greenhouse effect, between 35% and 65 % for clear sky conditions and between 65% and 85% for a cloudy day. Water vapor concentration fluctuates regionally and locally, whereas it is not directly affected by human activities. The addition of the non-condensable greenhouse gases causes the temperature to increase, leading to an increase in water vapor that further increases the temperature. This is the so-called positive feedback effect. If more water vapor is added to lead to more cloud formation, clouds reflect and block sunlight and reduce the amount of energy that reaches the Earth's surface to warm it. This is a negative feedback effect.

Increases in water vapor can be responsible for lower atmospheric and Earth's surface temperatures [3]. Their impacts have been studied using different computer simulations of global warming induced by different emission gases, from simple zero-dimensional model [4], one-dimensional model [4, 5, 6] to complex 3-dimensional, short period [7] to long periods [8], heat conduction to convection and radiation [5, 6], coupled with complicated ocean-atmosphere models [9]. However, one-dimensional models have advantages to reveal certain basic mechanisms and feedbacks of global warming [4]. More relevancy of the calculations, however, depends on the validity of the models with reliable and realistic radiative transfer. A rather detailed and systematical analysis accounting for radiative absorption and emission as function of temperature, pressure, concentration and wavelength is still needed.

A fundamental, systematical and detailed study of absorption of water vapor across the troposphere layer is required to solve the radiative transfer equations together with relevant values of radiative properties. There have many articles for measurements of absorption of water vapor in different bands [10, 11, 12, 13, 14, 15]. More simplicity for spectral modelling is the calculation of one value for every spectral band. These types of models are the wide band models. Excellent reviews can be found in Modest [16], Howell et al. [17], Edwards [18], Cess and Tiwari [19], Tien [20], etc. The total band absorption A was first measured extensively for different pressures and absorber concentration of carbon dioxide and water vapor at an atmospheric temperature by Howard et al. [15, 21]. It was found that the total band absorptance A would be linear with the density-path length product, X, at low values, $X^{1/2}$ at moderate values, and a logarithmic relation for highest experimental values. More accurate and simple wide-band models are thus constructed for theoretical bases [22, 23, 24, 25, 26, 27]. A further successful model for wide band absorption has been shown by Edwards and Menard [28] using the Goody model for spectral absorptivity to construct band models. The vibrating rigid rotator and the vibrating non-rigid rotator were employed to obtain the Goody parameters. Asymptotic relations for the total band absorption vs mass path length and pressure obtained from each model were thus successfully found to be of linear, square root, square root logarithmic, and logarithmic relations.

In this work, absorption coefficients of water vapor suitable for a heat transfer analysis in the troposphere atmosphere are systematically and quantitatively determined. To clarify the above-mentioned controversy, it is necessary to study absorption coefficients in different bands leading to a more realistic prediction of radiative transport and temperature profiles. This work provides a fundamental step for a more realistic and quantitative understanding of greenhouse effects related to global warming phenomena.

## 2 Model

**Fig. 2:**
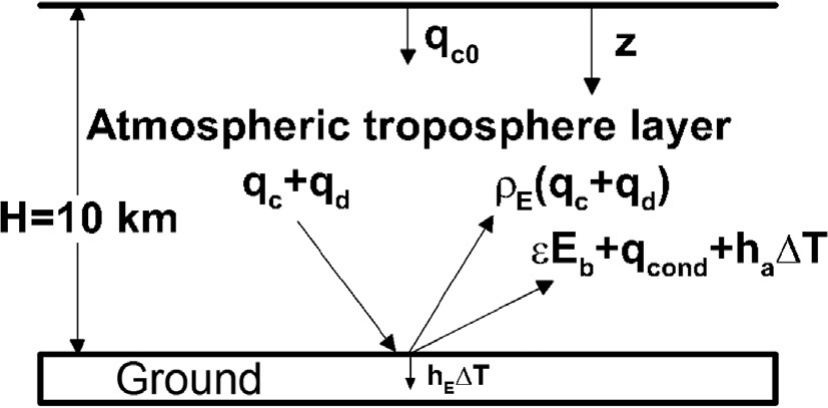
The physical model and coordinate system [1].

The physical domain proposed in this study is the troposphere layer on the Earth's surface, as illustrated in Fig. 2. Solar irradiation $q_{c0}$ imposed at the tropopause at z = 0 is absorbed, scattered and transmitted through the troposphere, and absorbed by the Earth's surface at z = H. Collimated and diffuse components of radiation, namely, $q_{c}$ and $q_{d}$, and their reflections and surface emissions are balanced by heat conduction and convection at the Earth's surface. The major assumptions made are the following:

1. The troposphere is a clear sky without clouds or rain or snow. All of these precipitations are required for water vapor to form.
2. The atmosphere is composed of air or nitrogen and oxygen, water vapor and carbon dioxide. Significant concentrations of symmetric diatomic molecules such as oxygen gas and nitrogen gas have no permanent dipole moment, resulting in transparency to infrared radiation.
3. Solar flux is governed by the Beer's law, whose extinction coefficient can be slightly modified by inclusion of Mie and Rayleigh scatterings [29, 30].
4. Absorption is resulting from water vapor and carbon dioxide. Absorption bands of water vapor are considered to be in wavelength ranges centered at 71, 6.3, 2.7, 1.87 and 1.38 μm [16,17]. Those of carbon dioxide are centered at 15, 4.3, 2.7, and 2 μm. Absorption coefficients are effectively evaluated from an exponential wide band model [16, 17, 18, 19, 20].
5. The diffuse component of radiative transfer is solved by the widely used $P_{1}$ approximation [16].
6. Heat transfer is unsteady and one-dimensional across the troposphere. This is attributed to thermal boundary layer thickness of the troposphere layer much thinner than the affected region of solar irradiation.
7. Heat transport affected by fluid flow near the Earth's surface can be accounted by $h_{a}\left(T-T_{ref}\right)$, where the heat transfer coefficient $h_{a}$ = 10 $W/m^{2}-K$, and reference temperature $T_{ref}$ = 289 K at location z = 9,900 m. Heat conduction into the Earth's ground is similarly evaluated by $h_{E}\left(T-T_{E\infty }\right)$, where heat transfer coefficient $h_{E}$ = 20 $W/m^{2}-K$, whereas reference temperature $T_{E\infty }$ = 286 K in the Earth's ground [31].
8. The Earth's ground is opaque to incident radiation. As a result, reflectivity $\rho_{E}$ = 1-ε, where ε is emissivity of the Earth's surface.

### 2.1 Absorption coefficients

Absorption coefficient of water vapor and carbon dioxide can be effectively determined by the exponential wide band model [1, 16, 17].

(1) $$\kappa =\frac{\alpha }{\Delta \eta }\approx \frac{\tilde{\alpha }\rho_{H_{2}O}}{\tilde{\omega }A^{*}}\text{,}$$

where α and Δη are band intensity and effective band width, $\tilde{\alpha }$ the correlation parameter and $\tilde{\omega }$ the correlation parameter related to band width, respectively. In this work, subscript of water vapor density $\rho_{H_{2}O}$ is eliminated for brevity. Three regions in each absorption band are defined by dimensionless total band absorptance $A^{*}$ with the following three relationships:

For β≤1

(2) $$A^{*}=\tau_{0}\;\text{for}\;0\leq \tau_{0}\leq \beta$$

(3) $$A^{*}=2\sqrt{\tau_{0}\beta }-\beta ,\;\text{for}\;\beta \leq \tau_{0}\leq \frac{1}{\beta }$$

(4) $$A^{*}=\ln\left(\tau_{0}\beta \right)+2-\beta ,\;\text{for}\;\frac{1}{\beta }\leq \tau_{0}<\infty$$

where dimensionless parameters $\tau_{0}$ and β are optical thickness at the band center and overlap parameters, respectively, given by

(5) $$\tau_{0}=\frac{\tilde{\alpha }\rho s}{\tilde{\omega }}\text{,}$$

(6) $$\beta \equiv \gamma P_{e}$$

Quantities s, γ and $P_{e}$ in Eqs. (5) and (6) are optical path length, correlation parameter and effective pressure due to collisions, respectively. Region 1 given by Eq. (2) is irrelevant in absorption in troposphere. This is because absorption coefficient in Region 1 by combining Eqs. (1), (2), and (5) is simplified to a reciprocal of selected optical path length, which is independent of radiation properties. Temperature or density-dependent correlation parameters in Eqs. (5) and (6) are

(7) $$\alpha ={\tilde{\alpha }}_{\text{0}}\left(\frac{\tilde{\alpha }}{{\tilde{\alpha }}_{\text{0}}}\right)\rho$$

(8) $$\tilde{\omega }={\tilde{\omega }}_{0}\sqrt{\frac{T}{T_{0}}}\;\text{,}\;\;{\text{T}}_{\text{0}}=100\;\text{K}$$

(9) $$\gamma \;={\tilde{\gamma }}_{\text{0}}\left(\frac{\gamma }{{\tilde{\gamma }}_{\text{0}}}\right)$$

where T is temperature, values of correlation parameters ${\tilde{\alpha }}_{\text{0}}$, ${\tilde{\omega }}_{\text{0}}$ and ${\tilde{\gamma }}_{\text{0}}$ are listed in [16, 17]. The effective pressure in Eq. (6) yields

(10) $$P_{e}\equiv {\left[\frac{P_{a}}{p_{0}}\left(1+\left(b-1\right)x\right)\right]}^{n},\;p_{0}=1\;atm$$

where $P_{a}$ and x are, respectively, total pressure of air and mole fraction of water vapor, b and n are pressure parameters [16, 17]. The variation of effective band width defined in Eq. (1) in each region with depthwise position leads to

(11) $$\frac{d\Delta \eta \left(\rho ,T\right)}{dz}=\tau_{0}\frac{dA^{*}}{d\tau_{0}}\frac{\tilde{\omega }}{\rho }\frac{d\rho }{dz}+\left(A^{*}-\tau_{0}\frac{dA^{*}}{d\tau_{0}}\right)\frac{d\tilde{\omega }}{dT}\frac{dT}{dz}$$

Substituting Eqs. (2), (3), and (4), the ratio between coefficients of Eq. (11) gives

(12) $$\frac{A^{*}-\tau_{0}\frac{dA^{*}}{d\tau_{0}}}{\tau_{0}\frac{dA^{*}}{d\tau_{0}}}=\{\begin{array}{l} 0\;\text{for}\;\tau_{0}\to \text{0}\;\\ \infty \;\text{for}\;\tau_{0}\to \infty \end{array}$$

Eqs. (11) and (12) therefore indicate that effective band width is dominated by water vapor density and temperature for small and large optical thicknesses at the band center, respectively.

### 2.2 Heat equations in atmosphere

The unsteady one-dimensional energy equation in the troposphere yields

(13) $$\frac{\partial \rho_{a}c_{p}T}{\partial t}=\frac{\partial }{\partial z}\left(k_{a}\frac{\partial T}{\partial z}\right)-\frac{\partial q_{c}}{\partial z}-\frac{\partial q_{d}}{\partial z}$$

where t, $\rho_{a}$, $c_{p}$, and $k_{a}$ are, respectively, time, density, specific heat and thermal conductivity of air. The term on the left-hand side represents change in enthalpy with time, whereas terms on the right-hand side are, respectively, heat conduction, and absorption of radiative energy due to collimated and diffuse components [16]. Energy balance at the Earth's surface is given by

(14) $$\;-k_{a}\frac{\partial T}{\partial z}=h_{E}\left(T_{E}-T_{g}\right)+h_{a}\left[T_{E}-T\left(9900,0\right)\right]-\left(1-\rho_{E}\right)\left(q_{c}+q_{d}\right)+\varepsilon_{E}\sigma T_{E}^{4}$$

where the term on the left-hand side represents heat conduction from the air to Earth's surface. Terms on the right-hand sides are, respectively, heat transfer into the ground, heat convection to the air, and absorption and emission of radiation by the Earth's surface. Factor of $1-\rho_{E}$ represents percentage of absorbed irradiation by the Earth's surface. The collimated component in the second term on the right-hand side of Eq. (13) governed by the Beer's law yields

(15) $$\frac{\partial q_{c}}{\partial z}=-\beta_{c}q_{c}$$

which $\beta_{c}$ is the extinction coefficient. Diffuse component in the last term of Eq. (13) is governed by [16].

(16) $$\frac{\partial {\text{q}}_{\text{d,ij}}}{\partial z}=\kappa_{ij}\left(4E_{b,j}-G_{d,ij}\right)$$

(17) $$\begin{array}{l} \frac{\partial G_{d,ij}}{\partial z}=-3\kappa_{ij}{\text{q}}_{\text{d,ij}}\\ \;\;\;\; \end{array}$$

where subscripts i and j represent water vapor or carbon dioxide and absorption band, and $E_{b,j}$ and $G_{d,ij}$ are emissive power of a blackbody and incident radiation function, respectively [16]. The total diffuse radiative flux including carbon dioxide and water vapor in different absorption bands thus yields

(18) $$q_{d}=\sum_{gas\;i,\;band\;\text{j}}q_{d,ij}\;\;=\sum_{band\;j}q_{d,CO_{2}j}+\sum_{band\;\text{j}}q_{d,H_{2}O\;j}$$

Boundary conditions of the diffuse radiation at the tropopause and Earth's surface are, respectively

(19) $$2q_{d,ij}=\frac{\varepsilon_{T}\left(4E_{b,j}-G_{d,ij}\right)}{2-\varepsilon_{T}}\;\text{at}\;z=0$$

(20) $$-2q_{d,ij}=\frac{\varepsilon \left(4E_{b,j}-G_{d,ij}\right)}{2-\varepsilon }\;\text{at}\;z=H$$

where radiative properties $\varepsilon_{T}$ and ε are emissivities at the tropopause and Earth's surface, respectively.

## 3 Results and discussion

In this work, absorption coefficient as a function of temperature, density and correlation parameters for radiative properties is predicted by using exponential wide band model. The COMSOL computer code with the Heat Transfer Module was utilized to solve one-dimensional unsteady heat transfer Eq. (13) with boundary condition of Eq. (14) and convection at the tropopause, whereas the PDE Module was used to solve collimated and diffuse radiation fluxes governed by Eqs. (15), (16), (17), (18), (19), and (20), where absorption coefficient is updated at each time. The following figures provided are at 6 am in one month after December 27. Initial temperature is considered linear across the troposphere. The temperature profile gradually becomes independent of the initial temperature as time increases.

### 3.1 Comparison with theoretical and experimental data

**Fig. 3:**
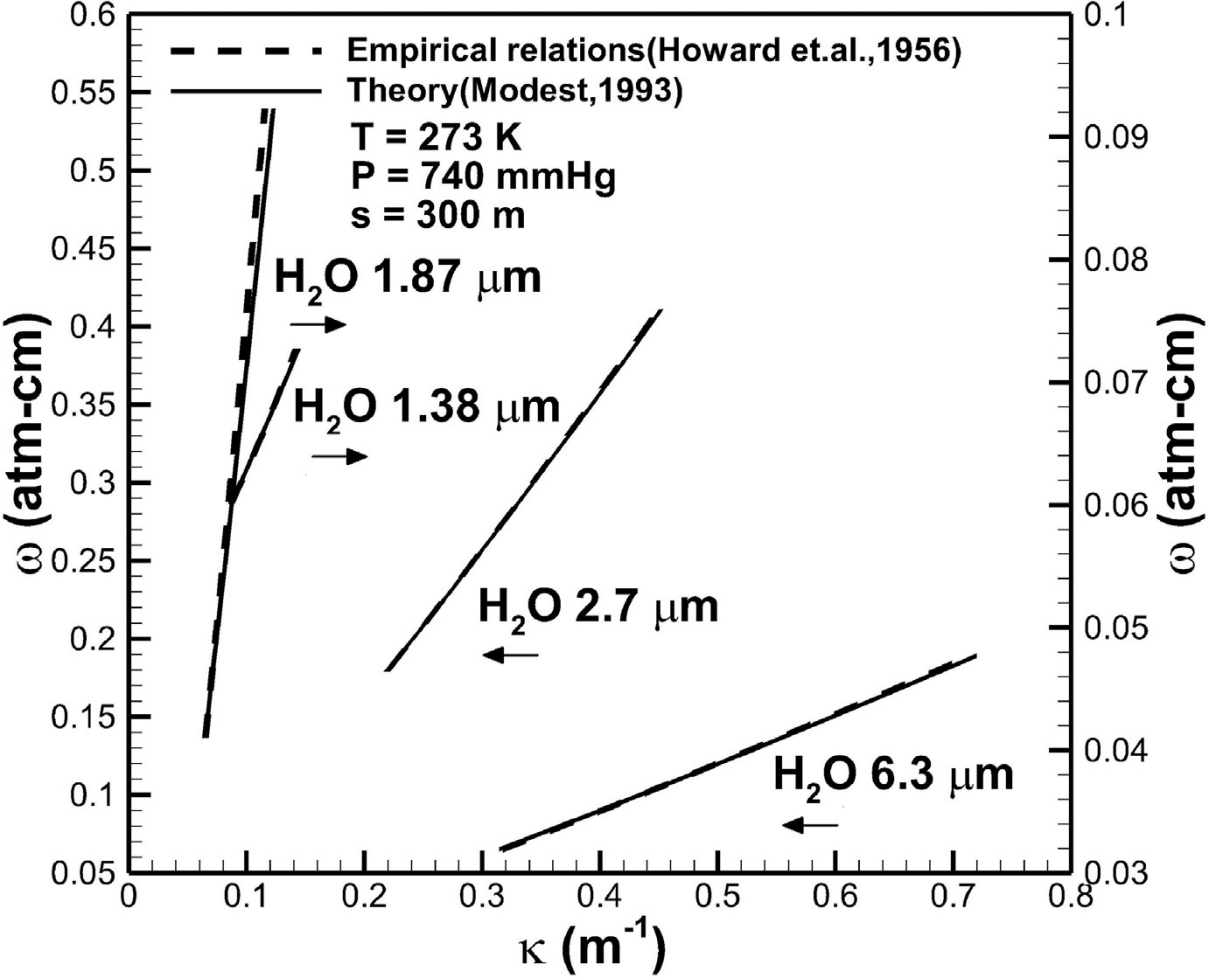
Comparison of absorption coefficients of water vapor in different bands predicted from available theory [15] and this work based on the exponential wide band model [16].

A good comparison between the predicted absorption coefficient of water vapor in different bands at a temperature of 273 K, total pressure of 740 mmHg, and optical path length of 300 m from this work using the exponential wide band model [16] and available correlation from experimental data [15] is shown in Fig. 3. The ordinates represent absorber concentration. It is a measure of the total number of absorbers per unit area traversed by the beam of radiation. According to the Avogadro's hypothesis the molecular weight M in grams in any gas occupies 22.4 liters and $6.02\times 10^{23}$ gas molecules at STP. Since 1 atm-cm is equivalent to a length of 1 cm of gas per unit $cm^{2}$, 1 atm-cm = $6.02\times 10^{23}/2.24\times 10^{4}$ = $2.69\times 10^{19}\;{\text{molecules/cm}}^{2}$ at STP. It is noted that the unit of concentration of water vapor from measurements [15] were usually expressed in terms of pr cm. For condensable water vapor, it is more convenient to measure the total length of liquid water in cm, which may be precipitated out of the path per unit area. The number of precipitable centimeter abbreviated by pr cm of water vapor in a given path of length is thus given by 1 pr cm for $H_{2}O$ = $6.02\times 10^{23}/M=3.34\times 10^{22}$ molecules/$cm^{2}$. The constant ratio between pr cm and atm-cm can be included into the constant “C” in the correlations equations provided by Howard et al. [15]. The comparison between the correlation equations and prediction from exponential wide model [16] can thus be made, provided that one point is chosen to be coincided. It can be seen that absorption coefficient is of the order of $0.1-1{\;\text{m}}^{-1}$. An increase in concentration of water vapor increases absorption coefficient. Similar agreements can be seen in wavelength bands centered at 2.7 μm, 1.87 μm, and 1.38 μm.

**Fig. 4:**
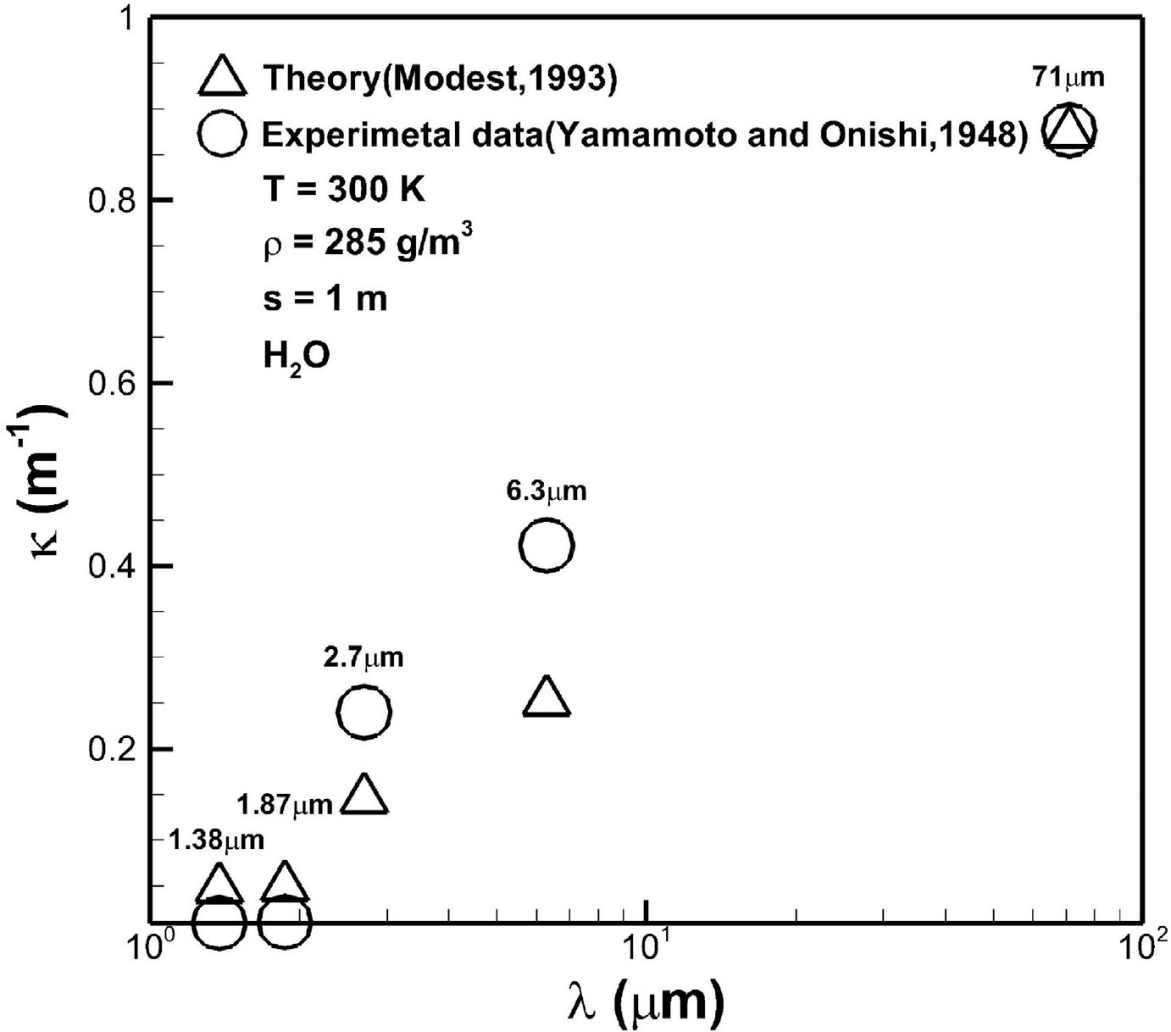
Comparison of averaged absorption coefficients in different bands of water vapor between predicted results from this work [16] and available measurements [32].

Fig. 4 shows a consistent comparison of averaged absorption coefficients in different bands of water vapor between predicted results from this work [16] and available measurements [32]. Aside from approximation induced by the exponential wide band model, one factor affecting deviations between theoretical and experimental results may be difficult in controlling of experimental conditions [33]. The critical optical path length, as can be seen later, was also uncertain. The first, second and third highest absorption coefficients are, respectively, referred to the bands centered at 71 μm and 6.3 μm and 2.7 μm. This is primarily attributed to magnitudes of correlation parameter of ${\tilde{\alpha }}_{\text{0}}$. These values also agree with theoretical values as shown in previous Fig. 3.

### 3.2 Absorption coefficient affected by optical path length

**Fig. 5:**
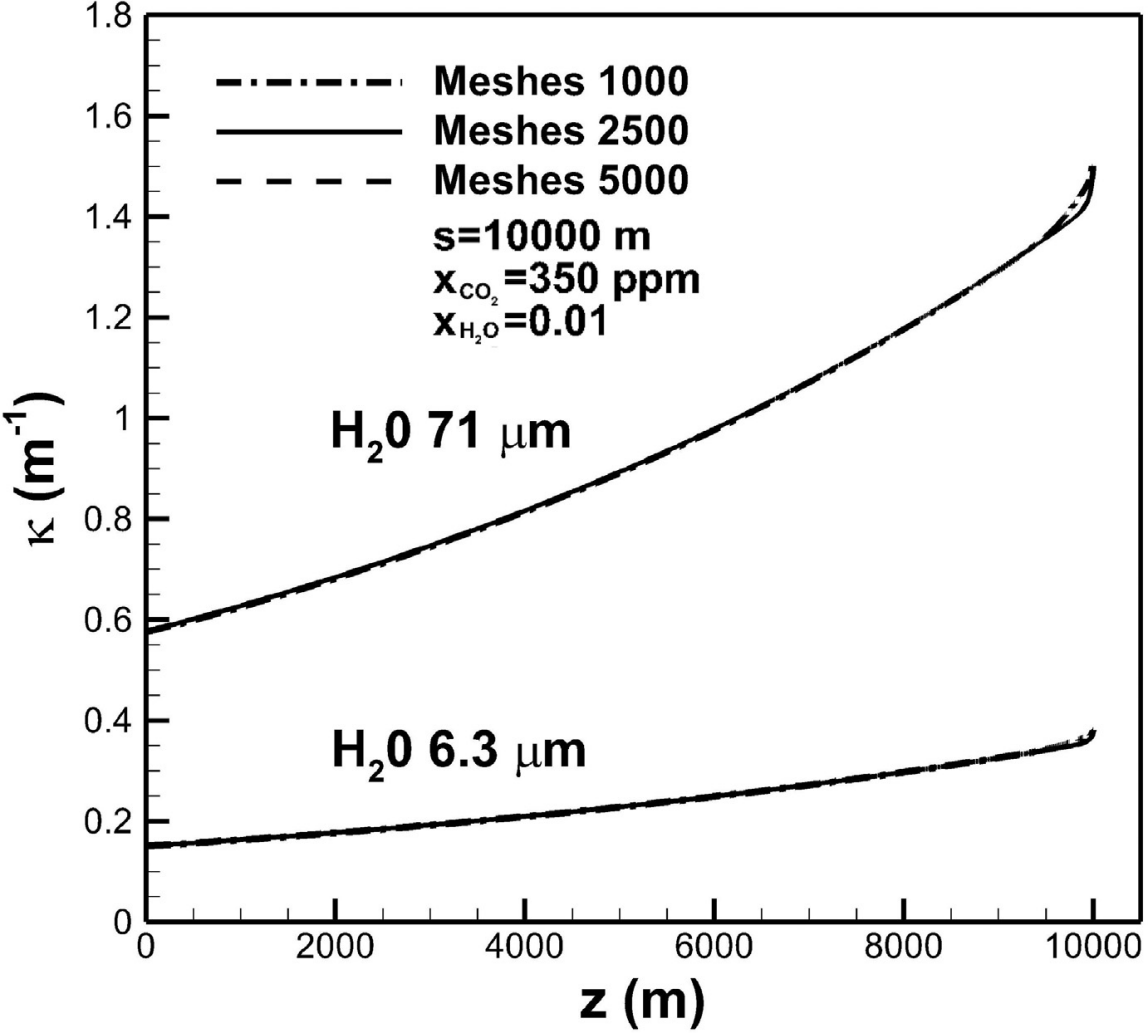
Convergence test of absorption coefficients in bands centered at 71 and 6.3 μm across the troposphere layer for different grid sizes.

**Fig. 6:**
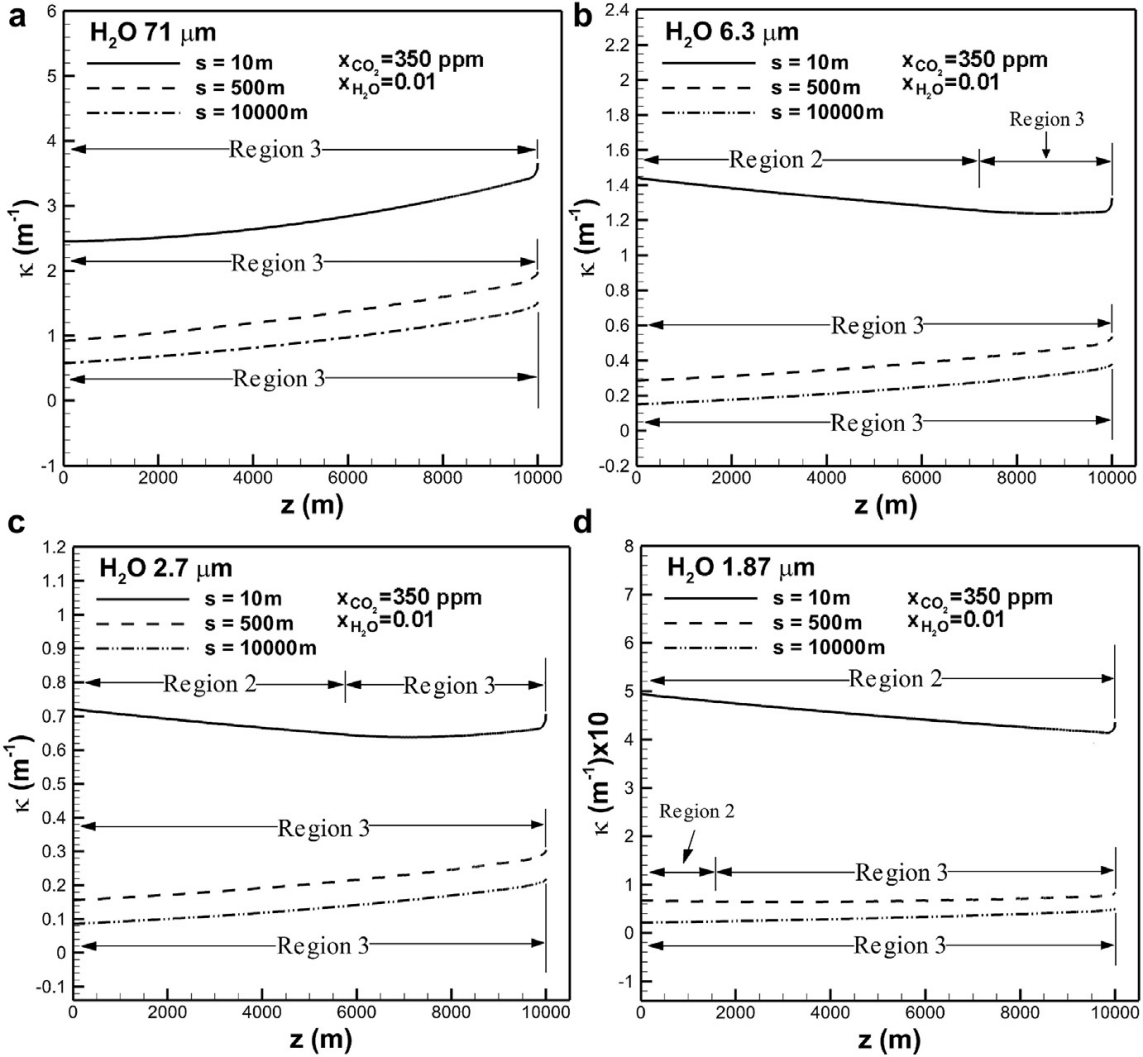
The effects of optical path length on absorption coefficient in different bands centered at (a) 71, (b) 6.3, (c) 2.7, and (d) 1.87 μm across the troposphere layer.

Fig. 5 shows convergence test of the first and second highest absorption coefficients of water vapor in bands centered at wavelengths of 71 and 6.3 μm across the troposphere layer for different grid sizes. A slight deviation can be seen near the Earth surface, provided that coarse meshes of 1000 are used. To assure convergence, dense meshes are selected near the Earth's surface. Meshes greater than 2,500 can thus give good results through the troposphere. Fig. 6(a) shows the effects of optical path length on absorption coefficient in the band centered at wavelength of 71 μm with mole fractions of water vapor and carbon dioxide of 0.01 and 350 ppm, respectively. The troposphere can be divided into layers in different optical path lengths to accurately predict absorption coefficient as a function of temperature, concentration and altitude. As mentioned previously, absorption coefficients depending on different optical thicknesses at band centers are categorized by three regions governed by Eqs. (2), (3), and (4), where the overlap parameter is usually smaller than unity in the troposphere. This is attributed to small values of correlation parameter ${\tilde{\gamma }}_{\text{0}}$ and partial pressure of water vapor (see Eqs. (6), (9), and (10)). Regardless of optical path lengths, only Region 3 governed by Eq. (4) is prevailed in the entire troposphere. High optical thickness at the band center is due to high value of correlation parameter ${\tilde{\alpha }}_{\text{0}}$ [16]. Physically speaking, it indicates that water vapor experiences nonrigid rotation in the troposphere [22, 28]. Absorption coefficient decreases from around 3.8 to 1.5 ${\text{m}}^{-1}$ near the Earth's surface, as optical path length increases from 10 to $10^{4}$ m.

Regions 2 and 3 can occur in the upper and lower regions of the troposphere in the case of bands centered at 6.3 and 2.7 μm for an optical path length of 10 m, as shown in Fig. 6(b) and (c), respectively. Occurrence of Region 2, indicating small optical thickness at the band center, in the upper region is attributed to low density of water vapor (see Eqs. (11) and (12)). Region 3, however, prevails in the entire troposphere for optical path length of 500 m and $10^{4}$ m. Fig. 6(d) shows that Regions 2 becomes prevailed in the case of absorption band centered at 1.87 μm and optical path length of 10 m. Regions 2 and 3 can be seen in the upper and lower regions of troposphere subject to optical path length of 500 m. Referring to Fig. 6(a), (b), (c) and (d) show that Region 3 prevails in the entire troposphere in all bands with high optical path length of $10^{4}$ m. The change in absorption coefficient decreases as the optical path length increases. Relevant values for the path length were chosen as high as around 5.5 km as suggested by Ceballos et al. [30], Smirnov [34] and Shaw [35]. A more accurate measurement of absorption coefficient through the troposphere is essentially required.

**Fig. 7:**
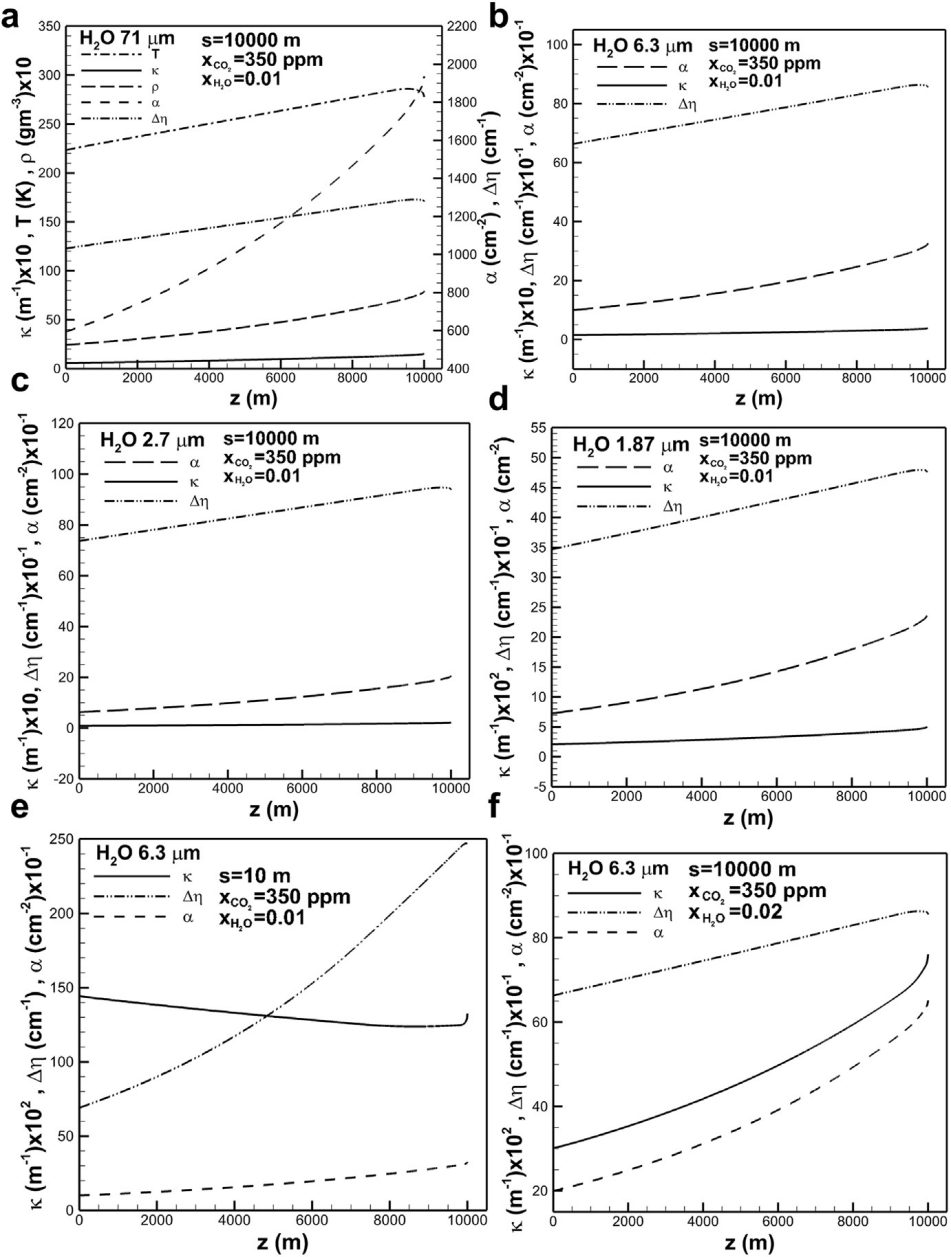
Absorption coefficient, temperature, density, band intensity and effective band width in band centered at (a) 71 μm, and absorption coefficient, band intensity and effective band width of bands centered at (b) 6.3 μm, (c) 2.7 μm, (d) 1.87 μm with mole fraction of 0.01 of water vapor and optical path length of $10^{4}$ m, (e) 6.3 μm with mole fraction of 0.01 of water vapor and optical path length of 10 m, and (f) 6.3 μm with mole fraction of water vapor of 0.02 and optical path length of $10^{4}$ m across the troposphere layer.

The variations of absorption coefficient, temperature, density, band intensity and effective width of the band centered at 71 μm for given optical path length of $10^{4}$ m, carbon dioxide concentration of 350 ppm and water vapor concentration of 0.01 across the troposphere are shown in Fig. 7(a). Rather than exponential increases in water vapor density and band intensity, temperature and effective band width increase approximately linearly and then decrease in the direction toward the Earth's surface. The increase in absorption coefficient as the Earth's surface is approached is attributed to the increase in band intensity to be greater than that of effective band width. Since temperature dominates effective band width (see Eqs. (11) and (12)) in a high optical thickness at the band center, a comparatively rapid increase in absorption coefficient in the direction toward and near the Earth’ surface is therefore attributed to not only a rapid increase in band intensity but also slightly decrease in effective band width affected by temperature. Figs. 7(b), (c) and (d) shows similar trends of absorption coefficients, band intensity and effective band width of absorption band centered at 6.3, 2.7 and 1.87 μm, respectively. In view of heat equation accounting for all absorption bands, the temperature profile is the same as that shown in previous Fig. 7(a). Fig. 7(e) show absorption coefficients, band intensity and effective band width of absorption band centered at 6.3 μm with an optical path length of 10 m. In view of small optical path length leading to a small optical thickness at the band center, effective band width is dominated by water vapor density (see Eqs. (11) and (12)). An exponential increase in water density results in a similar increase in effective band width. Absorption coefficient decreases and then increases in the direction toward the Earth's surface. With optical path length of $10^{4}$ m, the effects of water vapor density on absorption coefficient, band intensity and effective band width are shown in Fig. 7(f). Referring to previous Fig. 7(b) it shows that an increase in mole fraction of water vapor from 0.01 to 0.02, absorption coefficient increases from around 0.2 to 0.75 ${\text{m}}^{-1}$ near the Earth's surface. Effective band width is insensitive to the variation of water vapor concentration.

### 3.3 Absorption coefficient affected by water vapor concentration

**Fig. 8:**
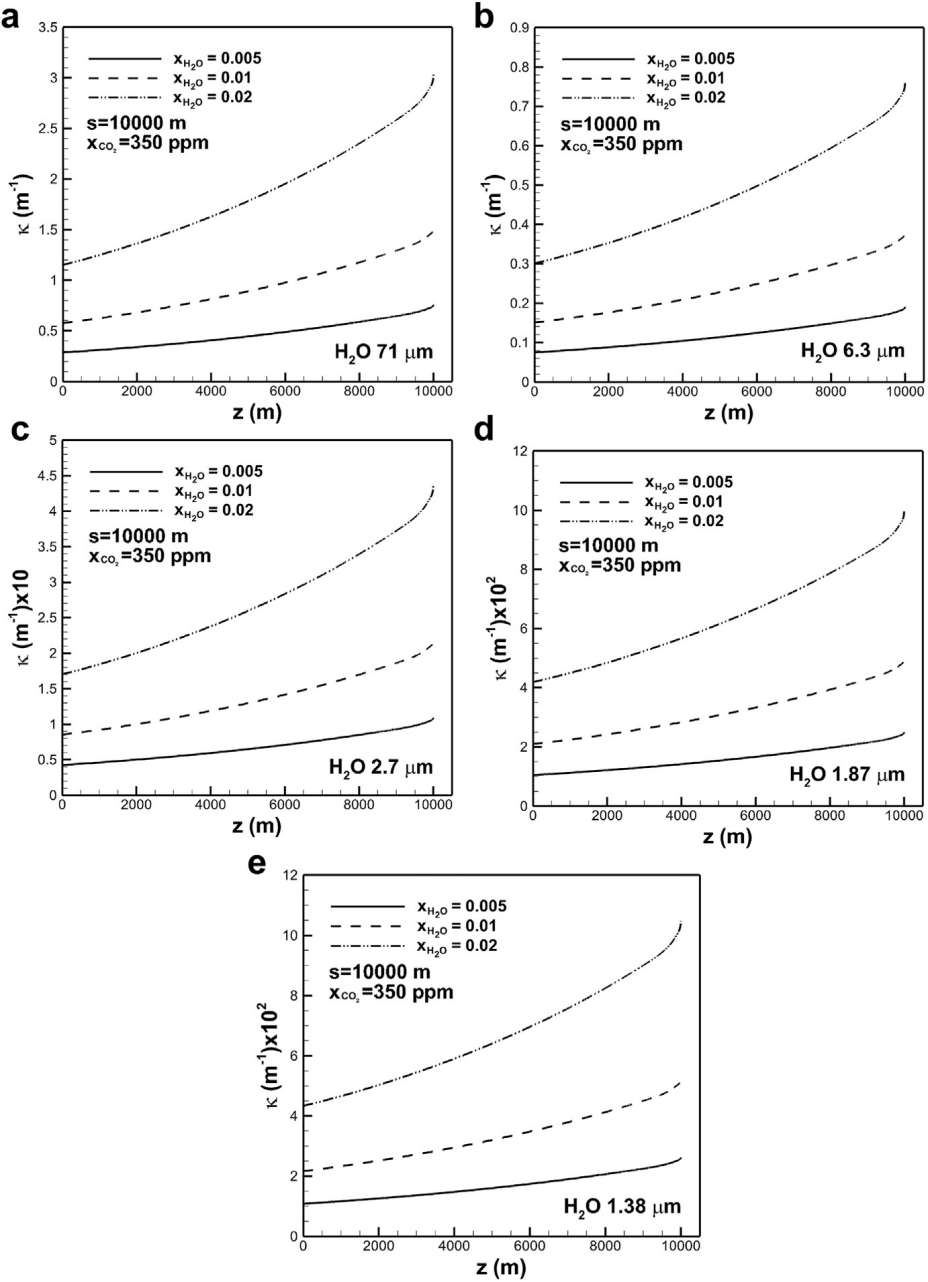
The effects of mole fraction of water vapor on absorption coefficient across the troposphere in different bands centered at (a) 71, (b) 6.3, (c) 2.7, (d) 1.87 and (e) 1.38 μm.

The effects of mole fraction of water vapor on absorption coefficient subject to optical path length of $10^{4}$ m in the band centered at 71 μm, and carbon dioxide concentration of 350 ppm are shown in Fig. 8(a). An increase in mole fraction of water vapor significantly increases absorption coefficient. This is attributed to an increase in band intensity, which the product of density and correlation parameter $\tilde{\alpha }$ of water vapor. For a mole fraction of water vapor of 0.005, absorption coefficient increases from around 0.3 ${\text{m}}^{-1}$ at the tropopause to 0.6 ${\text{m}}^{-1}$ at the Earth's surface. For a mole fraction of water vapor of 0.02 absorption coefficient increases from around 1.15 ${\text{m}}^{-1}$ at the tropopause to 3.1 ${\text{m}}^{-1}$ at the Earth's surface. The trends of absorption coefficients through the troposphere for different water vapor concentrations are similar. The difference of absorption coefficient across the troposphere therefore increases with water vapor concentration. Fig. 8(b) shows that absorption coefficients increase from 0.08 ${\text{m}}^{-1}$ and 0.3 ${\text{m}}^{-1}$ at the tropopause to 0.15 ${\text{m}}^{-1}$ and 0.78 ${\text{m}}^{-1}$ at the Earth's surface for mole fraction of water vapor of 0.005 and 0.02, respectively, in the band centered at 6.3 μm. Absorption coefficients of band centered at 2.7, 1.87 and 1.38 μm exhibit similar trends, as shown in Fig. 8(c), (d) and (e). Absorption coefficients decrease with wavelengths at band centers.

### 3.4 Temperature profile across tropospheric layer

**Fig. 9:**
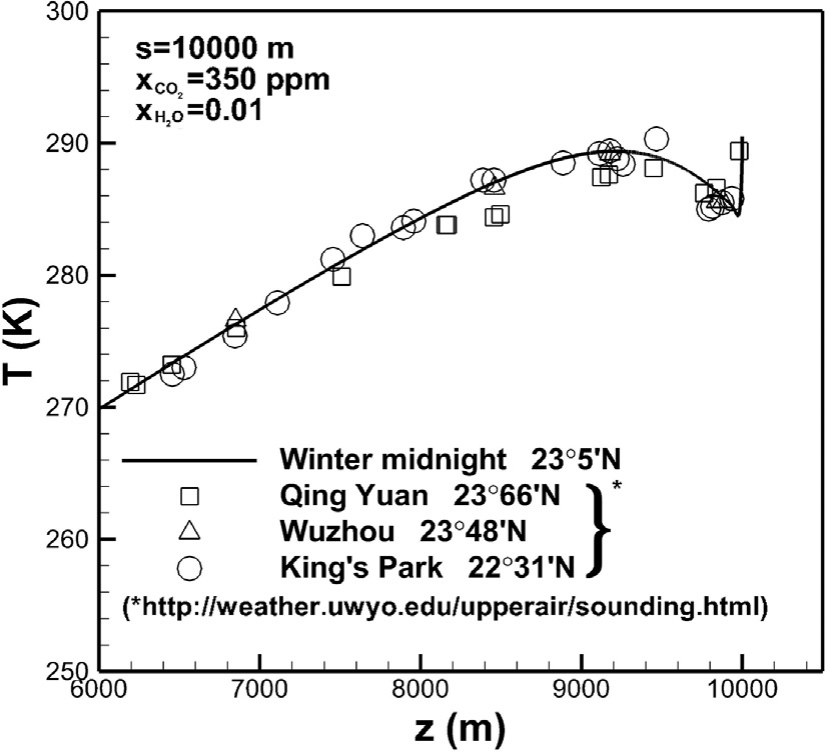
Comparison of a temperature profile across the troposphere between experimental data and this work.

A good and detailed comparison of a temperature profile across the troposphere at the noon of winter on Dec. 26 between experimental data and this work is shown in Fig. 9. Three locations are approximately at identical latitude. In this case, reference temperature and heat transfer coefficient in the ground $T_{E\infty }$ = 286 K and $h_{E}$ = 5 $W/m^{2}-K$. In the air, the reference temperature $T_{ref}$ = 299 K and heat transfer coefficient

(21) $$h_{a}=h_{a1}\left[\frac{1}{2}\left(\sin\frac{\pi t}{t_{d}}+\left|\sin\frac{\pi t}{t_{d}}\right|\right)\right]-h_{a2}\left[\frac{1}{2}\left(\sin\frac{\pi t}{t_{d}}-\left|\sin\frac{\pi t}{t_{d}}\right|\right)\right]$$

where one daily time $t_{d}$ = 43,200 s. In Eq. (21) heat transfer coefficient in daytime $h_{a1}$ = 5 $W/m^{2}-K$, whereas that in the evening $h_{a2}$ = 500 and 40$W/m^{2}-K$ before and after Aug. 31, respectively. A local minimum and maximum take place near the location around 5 and 9,200 meters, respectively. Temperature exhibits the maximum value at the Earth's surface. This is attributed to strong absorption of solar irradiation at noon.

## 4 Conclusions

With the predicted temperature which can increase and then decrease, whereas water vapor density exponentially increases in the direction toward the Earth's surface, the conclusions drawn are the following:

1. Absorption coefficients as functions of concentration, temperature, optical path length, and correlated parameters of water vapor in different wavelength bands required for predicting temperature across the troposphere layer are rigorously and quantitatively investigated.
2. Absorption coefficient representing the ratio between band intensity and effective band width increases in the direction toward the Earth's surface. A rapid increase occurs near the Earth's surface. The increase in band intensity is dominated by an exponential increase in water vapor density. Effective band width for a high optical path length increases and then decreases to follow the trend of temperature.
3. Effective band width is dominated by water vapor density and temperature for small and large optical thicknesses at the band center, respectively. Optical thickness at the band center is proportional to optical path length. Effective band width therefore exhibits exponential increases for a small optical path length in the direction toward the Earth's surface. For a large optical path length effective band width exhibits a linear increase and then decrease in the direction toward the Earth's surface.
4. Absorption coefficient and its variation decrease as optical path length increases.
5. Relevant values of optical path length should be beyond 5000 m in different bands. Water vapor thus experiences non-rigid rotation. The variation of absorption coefficient with optical path length is slight.
6. The first, second and third highest absorption coefficients are, respectively, for bands centered at 71, 6.3, and 2.7 μm.
7. Absorption coefficient in different bands increases significantly with water vapor concentration. Absorption coefficients in the band centered at 71 μm increases from 0.3 to 1.2 ${\text{m}}^{-1}$ at the tropopause and 0.6 to 3.1 ${\text{m}}^{-1}$ at the Earth's surface as mole fraction of water vapor increases from 0.005 to 0.02.
8. Experimental determination of optical thickness or optical path length is required in order to obtain more accurate absorption coefficient.
9. The unsteady, one-dimensional model proposed can provide a first step to clearly reveal the critical factors affecting complicated issues of global warming, even though unsteady but complicated two- and three-dimensional models are more relevant.

## Declarations

### Author contribution statement

Peng-Sheng Wei: Conceived and designed the experiments; Analyzed and interpreted the data; Wrote the paper.

Hsuan-Han Chiu, Chieh Lee, Yi-Cheng Tsai, Te-Chuan Ting: Conceived and designed the experiments; Performed the experiments; Analyzed and interpreted the data; Contributed reagents, materials, analysis tools or data.

Yin-Chih Hsieh, Da-Lun Yen: Conceived and designed the experiments; Performed the experiments; Analyzed and interpreted the data.

### Funding statement

This research did not receive any specific grant from funding agencies in the public, commercial, or not-for-profit sectors.

### Competing interest statement

The authors declare no conflict of interest.

### Additional information

No additional information is available for this paper.
